# The Electric Field Guided HaCaT Cell Migration Through the EGFR/p38 MAPK/Akt Pathway

**DOI:** 10.3390/cimb47010016

**Published:** 2024-12-31

**Authors:** Huajian Zhou, Shihao Zhang, Xiaoli Jin, Chunxian A, Peng Gong, Sanjun Zhao

**Affiliations:** School of Life Sciences, Yunnan Normal University, Kunming 650500, China; zhj2506797225@163.com (H.Z.); ah17607105742@163.com (S.Z.); 18809421992@163.com (X.J.); 18314138923@163.com (C.A.)

**Keywords:** electric field, cell migration, EGFR, p38 MAPK, Akt

## Abstract

Previous studies have shown that the endogenous electric field (EF) is an overriding cure in guiding cell migration toward the wound center to promote wound healing, but the mechanism underlying is unclear. In this study, we investigated the molecular mechanism of electric field-guided cell migration in human keratinocyte HaCaT cells. Our results showed that HaCaT cells migrate toward the anode under EFs. The phosphorylation levels of p38 MAPK and Akt were obviously elevated in the EF. Knocking down p38 MAPK obviously abolished directed migration of HaCaT cells under the EFs. Inhibiting p38 MAPK by SB203580 impaired the EF-guided cell migration. The electric field may guide HaCaT cell migration through the EGFR/p38 MAPK/Akt pathway.

## 1. Introduction

Keratinocytes are important components of the human skin epidermis, and the directional migration of keratinocytes is essential for skin wound healing. Experimental data have demonstrated that the endogenous electric field (EF) is an overriding cure in guiding cell migration to promote wound healing [1]. Several applications of the electric field in wound healing have been developed in the clinic, especially for accelerating chronic refractory wound healing, while the molecular mechanisms underlying electric field-guided cell migration are not well understood [2,3].

The p38 MAPK (mitogen-activated protein kinase) signaling pathway allows cells to interpret a wide range of external signals and respond appropriately by generating diverse biological effects [4]. Previous studies have shown that p38 MAPK is associated with the migration of vascular stem/progenitor cells [5], non-small cell lung cancer cells [6], and breast cancer cells [7].

In addition, Akt (a downstream signaling molecule of the PI_3_ kinase pathway) is also involved in diverse cell behaviors [8,9]. EFs may induce Akt activation in diverse cells [10]. Studies of human renal tubular epithelial cells have shown that their migration direction and speed in EFs were significantly inhibited after treating cells with inhibitors of p38 MAPK and Akt, respectively [11], and that the p38 MAPK/Akt signaling axis may regulate the migration of vascular smooth muscle cells [12].

Moreover, EGFR signaling has significant physiological functions [13], and EGFR phosphorylation may initiate multiple downstream cascades, including the parallel protein kinase B (Akt) and MAPK pathways [14]. Previous studies showed that EFs are able to orientate the redistribution of EGFR on the surface of breast cancer cell membranes toward the anode [15] and polarize the EGFR of mesenchymal stem cells [16] and keratinocytes [17] toward the cathode. The above evidence suggests that EFs may transmit signals into cells via EGFR, but the signaling transduction pathway underlined is not well understood. In this study, the immortalized human keratinocyte HaCaT cells, which are widely used in dermatology studies, were applied as the model in exploring the mechanism of electric field-guided cell migration. The role of p38 MAPK in electric field-guided cell migration was explored by both RNAi and specific inhibitors. The signaling transduction pathway among EGFR, p38 MAPK, and Akt were discussed as well. Our results demonstrated that the p38 MAPKs play an important role in EF-guided cell migration and the electric field may guide HaCaT cell migration through the EGFR/p38 MAPK/Akt pathway.

## 2. Materials and Methods

The electric field-guided cell migration was analyzed by a microscopy image system. RNAi as well as a chemically functional antagonist were applied to explore the function of p38 MAPK in electric field-guided cell migration. The expression and activation of related proteins after EF stimulation were analyzed by western bolt to explore the signaling transduction pathway involved in electric field-guided cell migration.

### 2.1. Cell Culture

The human immortalized keratinocyte cell line HaCaT (Kunming Cell bank, CAS, Cat. KCB200442YJ, Kunming, China) was obtained from Kunming Cell Bank, Chinese Academy of Sciences. The cells were cultured in high-glucose Dulbecco’s Modified Eagle’s Medium (DMEM) supplemented with 10% (*v*/*v*) fetal bovine serum (FBS) (ExCell Bio, FND500, Beijing, China) and 1% (*v*/*v*) antibiotic mixture (10,000 U/mL penicillin G and 10,000 μg/mL streptomycin) (BasalMedia, S110JV, Shanghai, China). The cells were maintained at 37 °C in a humidified incubator under 5% CO_2_ and regularly tested for contamination.

### 2.2. Inhibitors

SB203580 (HY-10256, MCE, Shanghai, China), the inhibitor of p38 MAPK, was diluted to 10 mM in DMSO and finally diluted to 30 μM in HaCaT culture medium. The Akt inhibitor MK2206 (2HCl) (MCE, HY-10358, Shanghai, China) was diluted to 10 mM in DMSO and finally diluted to 10 μM in HaCaT culture medium. The EGFR inhibitor AG1478 (Selleck, S2728, Shanghai, China) was diluted to 10 mM in DMSO and finally diluted to 10 μM in HaCaT culture medium. HaCaT cells were firstly incubated with or without inhibitor or no *n* for 1 h before electric field treatment.

### 2.3. siRNA Transfection

p38 MAPKα siRNA and si-negative control (siNC) were purchased commercially (General Biol Company, Chuzhou, China). For siRNA transfection, HaCaT cells were cultured in medium without antibiotic mixture. Before transfection, cells were washed twice in PBS, and then the Lipofectamine 2000 Reagent (Invitrogen, 1274436, Waltham, CA, USA) was used to transfect 100 nM siRNA into HaCaT cells according to the manufacturer’s protocol. Opti-MEM (Gibco, 31985-070, Grand Island, NY, USA) was used to culture the transfected cells, after which the medium was replaced with normal culture medium after 6 h of transfection. The sequences of the RNAi were as follows: siNC, 5′-UUCUCCGAACGUGUCACGUTT-3′; si-p38α, 5′-CCAAAUUCUCCGAGGUCUATT-3′.

### 2.4. Electrotaxis Assay

Electric field-guided cell migration was performed according to previous methods [18]. HaCaT cells were seeded in Boyden chambers at 1.0 × 10^5^ cells/dish on a fibronectin-coated area for 12 h, which allowed them to settle down before direct current electric field (dcEF) treatment. A cover glass was applied on the top of the chamber, which was sealed with high-vacuum silicone grease (Dow Corning, NSF/ANSI 51/61) around the chamber. The final space of the chamber through which the electric current passed was 22 × 10 × 0.3 (mm). A strength of 100 mV/mm dcEF, which is similar to the physiological strength of the endogenous EF generated during wound healing [19,20], was applied along the agar–salt bridges with kryptol electrodes via Steinberg’s solution. The multifields time-lapse image microscope (Carl Zeiss, Axiovert 100, Oberkochen, Germany) was used to record cell migration.

### 2.5. Western Blot Assay

Total protein was extracted from cells by using precooled RIPA buffer (MA0151, Meilunbio, Dalian, China), and the protein concentration was determined with a BCA protein assay kit (Beyotime Biotechnology, P0010S, Shanghai, China). The proteins were resolved on 10% SDS polyacrylamide gels (Beyotime Biotechnology, P0012A, Shanghai, China) and transferred onto PVDF membranes (Merck, IPVH00010, Kenilworth, NJ, USA), which were then incubated with primary antibodies, including the following: rabbit anti-EGFR antibody (Cell Signaling Technology, 4267, Boston, MA, USA), rabbit anti-p-EGFR antibody (2234, Cell Signaling Technology Boston, MA, USA), rabbit anti-p38 MAPK antibody (Cell Signaling Technology, 8690, Boston, MA, USA), rabbit anti-p-p38MPK antibody (Cell Signaling Technology, 4511, Boston, MA, USA), rabbit anti-Akt antibody (Cell Signaling Technology, 4691, Boston, MA, USA), rabbit anti-p-Akt antibody (Cell Signaling Technology, 9271, Boston, MA, USA), rabbit anti-β-tubulin antibody (Boster Bioengineering Co. Ltd., A01857-1, Wuhan, China), and rabbit anti-GAPDH antibody (Yeasen, 30202ES60, Shanghai, China). Next, the PVDF membranes were incubated with HRP Goat anti-Rabbit IgG secondary antibody (Huabio, HA1001, Hangzhou, China) for 1 h at room temperature. The protein bands were visualized using enhanced chemical luminescence reagent (Biosharp, BL520B, Hefei, Anhui, China). The expression levels of these proteins were normalized with GAPDH/β-tubulin.

### 2.6. Statistical Analysis

Data were obtained from at least three independent experiments and displayed as mean ± s.e.m. Student’s *t*-test was used to analyze differences between two groups with SPSS 20.0 software. *p* < 0.05 was considered statistically significant. The GraphPad Prism 8 software was used to plot all the graphs.

The Usiigaci automatic cell tracking system combined with ImageJ software was used to analyze the migration of HaCaT cells in the dcEF [21]. Four parameters were used to quantify the migration of cells. Directedness = ∑cosθ/*n*, where θ is the angle between the direction of the electric field and the trajectory of cell migration. The value of cosθ of 1 or –1 indicates that the migration direction of cells is parallel to the electric field, and a value of cosθ of 0 indicates that the trajectory of cells is perpendicular to the electric field. Track speed = cell movement distance/total time, and velocity (displacement) = cell movement displacement/total time. Euclidian persistence = the total distance/displacement of the cell movement, where a higher value indicates a stronger ability of the cell to maintain movement in a certain direction.

## 3. Results

### 3.1. HaCaT Cells Migrated Toward the Anode in dcEF

**Figure 1:**
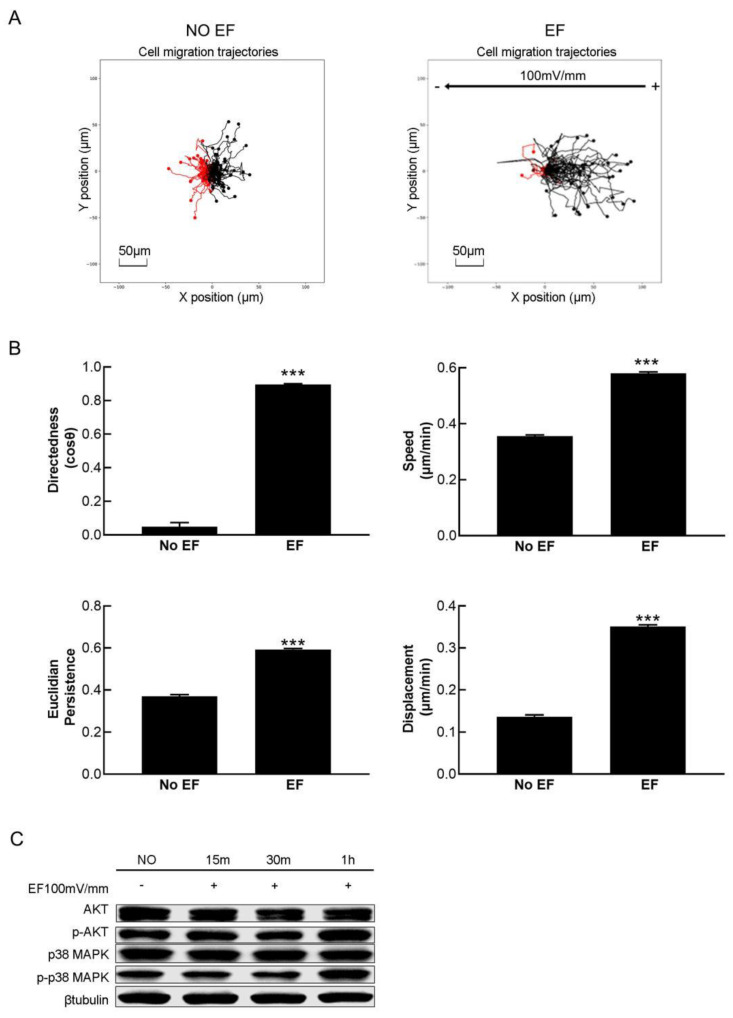
HaCaT cell electrotaxis and increased phosphorylation levels of p38 MAPK and Akt. (**A**) The migration trajectory of HaCaT cells under a 100 mV EF (*n* = 200). (**B**) HaCaT cell migration direction (cosθ), speed (μm/min), and Euclidian persistence and displacement (μm/min) in the EF (*n* = 200). (**C**) The phosphorylation levels of p38 MAPK and Akt were increased in the EF group. The data were obtained from three independent experiments and are presented as the mean ± s.e.m. *** *p* < 0.001 (two-tailed unpaired Student’s *t*-test).

HaCaT cells migrated randomly without EF stimulation, while they migrated toward the anode obviously in 100 mV/mm dcEF (Figure 1A). The migration directedness, speed, Euclidian persistence, and velocity of HaCaT cells were significantly increased under dcEF treatment (Figure 1B). Western blot analysis showed that the phosphorylation levels of p38 MAPK and Akt were obviously increased after EF stimulation, especially after 1 h of treatment (Figure 1C).

### 3.2. Inhibition of p38 MAPK Suppressed EF-Guided HaCaT Cell Migration

**Figure 2:**
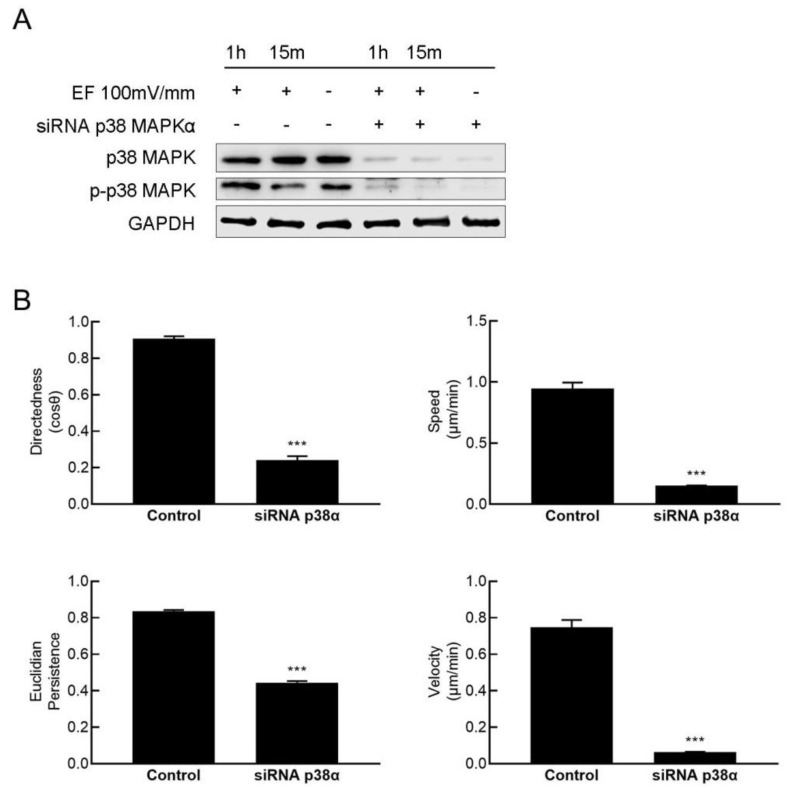
Knockdown of p38 MAPK obviously abolished the EF-guided cell migration. (**A**) Western blotting confirmed the knockdown of p38 MAPK by RNAi. (**B**) Knocking down p38 MAPK obviously abolished EF guided HaCaT cell migration (*n* = 300). The data were obtained from three independent experiments and are presented as the mean ± s.e.m. *** *p* < 0.001 (two-tailed unpaired Student’s *t*-test).

To study the role of p38 MAPK in electric field-guided HaCaT cell migration, p38α was knocked down by RNAi. Western blotting confirmed that the expression and phosphorylation of p38 MAPK were significantly interfered by RNAi (Figure 2A). Depletion of p38 MAPK by siRNA significantly abolished the migration of HaCaT cells in the EF. The migration directedness, velocity, track speed, and Euclidian persistence were significantly decreased compared to those of the control (Figure 2B).

**Figure 3:**
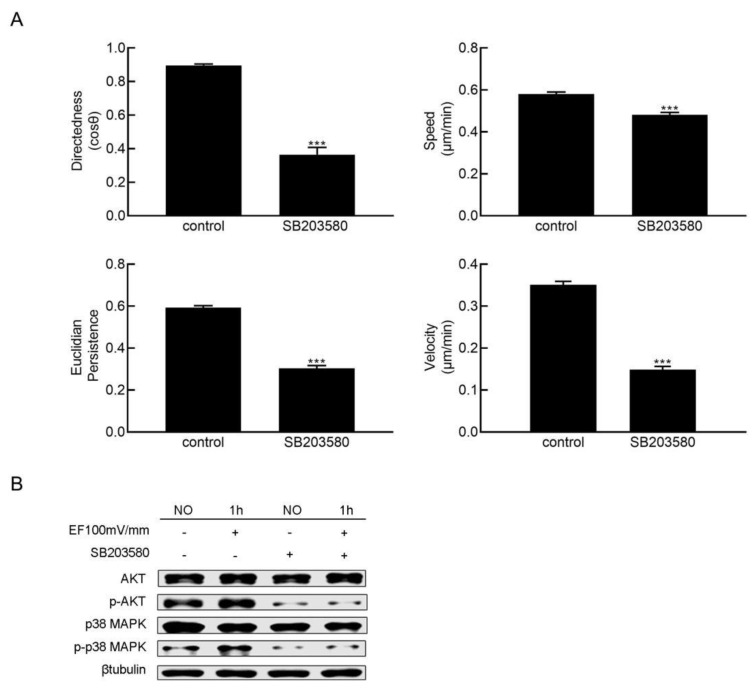
Inhibition of p38 MAPK impaired EF-guided HaCaT cell migration. (**A**) The migration direction (cosθ), speed (μm/min), Euclidian persistence, and velocity (μm/min) of HaCaT cells were obviously impaired by treatment with SB203580 (*n* = 300). (**B**) The phosphorylation of Akt was obviously impaired when the activation of p38 MAPK was inhibited by SB203580. The data were obtained from three independent experiments and are presented as the mean ± s.e.m. *** *p* < 0.001 (two-tailed unpaired Student’s *t*-test).

Treating HaCaT cells with the p38 MAPK inhibitor SB203580, the directedness, Euclidian persistence, velocity, and the migration speed of HaCaT cells in the EF were obviously reduced (Figure 3A). Western blotting showed that SB203580 treatment obviously inhibited the phosphorylation of p38 MAPK and Akt in HaCaT cells (Figure 3B).

### 3.3. The Electric Field Guided HaCaT Cell Migration Through the EGFR/p38 MAPK/Akt Pathway

**Figure 4:**
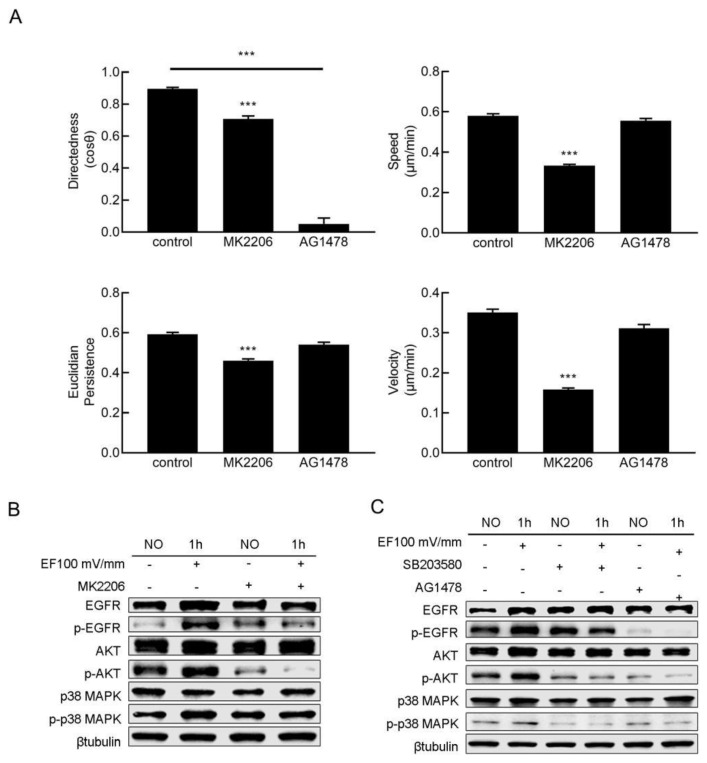
The electric field guided HaCaT cell migration through the EGFR/p38 MAPK/Akt pathway. (**A**) Blocking EGFR (AG1478) and Akt (MK-2206) impaired EF guided HaCaT cell migration (*n* = 300). (**B**) Blocking the activation of Akt with MK-2206 had no effect on the activation of EGFR or p38 MAPK. (**C**) Blocking EGFR resulted in decreased phosphorylation of p38 MAPK and Akt. Blocking p38 MAPK resulted in a decreased phosphorylation level of Akt. The data were obtained from three independent experiments and are presented as the mean ± s.e.m. *** *p* < 0.001 (two-tailed unpaired Student’s *t*-test).

To further understand the signaling transduction pathway of p38 MAPK in electric field-guided cell migration, the roles of EGFR and Akt in the p38 MAPK signal transduction pathway were studied with inhibitors. Considering that EGFR phosphorylation reportedly initiates multiple downstream cascades, including paralleled protein kinase B (Akt) and MAPK pathways [14], EGFR may act as an upstream regulator of MAPK and Akt. We first treated the cells with the EGFR inhibitor AG1478 (10 uΜ). The electric field-guided cell migration, especially the directedness, was obviously decreased (Figure 4A). The expression levels of p38 MAPK and Akt did not change upon AG1478 treatment, while the phosphorylation of EGFR, p38 MAPK, and Akt was markedly inhibited (Figure 4C). After the cells were treated with the p38 MAPK inhibitor SB203580, the expression and phosphorylation of EGFR showed no obvious changes (Figure 4C), while the phosphorylation level of Akt obviously decreased. After treating the cells with the Akt inhibitor MK2206, the HaCaT cells showed marked decreases in directedness, velocity, speed, and Euclidian persistence in response to EF stimulation (Figure 4A). Western blot analysis showed that MK2206 treatment decreased the phosphorylation of Akt, whereas the expression and phosphorylation of EGFR and p38 MAPK showed no obvious changes (Figure 4B). These results implied that the EGFR is the upstream of the signaling transduction pathway in the EF-guided cell migration and the EGFR/p38 MAPK/Akt signal transduction axis is involved in EF-guided cell migration.

## 4. Discussion

The mitogen-activated protein kinase (MAPK) family plays a crucial role in transmitting extracellular stimuli and results in a variety of intracellular responses, such as cell proliferation, differentiation, senescence and apoptosis, as well as migration [22,23]. Among them, ERK is mostly reported to be involved in EF-induced cell migration [24,25,26]; however, how p38 activation responds to EF stimulation is controversial [27]. Previous studies have shown that the p38 MAPK pathway and its downstream transcription factor activator protein-1 (AP-1) are negatively modulated by the EF in lipopolysaccharide (LPS)-induced inflammatory responses [28]. However, our study together with others showed the activation of p38 MAPK in the electric field. Furthermore, an increase in the phosphorylation level of p38 MAPK with increasing EF strength in a time-dependent manner was reported [11,29]. When myosin light chain kinase (myosin light chain kinase, MLCK) was inhibited in hepatocellular carcinoma cells, both p38 MAPK phosphorylation and E-calmodulin expression were elevated, and cell migration was significantly inhibited [30]. Furthermore, our experimental results showed that knocking down p38 MAPK abolished the electric field-guided HaCaT cell migration. Inhibition of p38 MAPK also resulted in a significant reduction in directed cell migration in electric fields, especially the directedness. Our results indicate that p38 MAPK is involved in sensing extracellular electrical stimuli and transmitting electrical signals, thus resulting in cell migration through electric fields in HaCaT cells.

EGFR, a member of the receptor family on the cell membrane, is activated, polarized, and redistributed in cells in the migration direction via EFs [15,17,31]. EGFR kinase activity and redistribution in the plasma membrane are required for the directional migration of keratinocytes in the EF [31]. Our results showed that inhibition of EGFR abolished the directed migration of HaCaT cells in the EF, while it had no obvious influence on the velocity of cell migration. Furthermore, the inhibition of EGFR, p38 MAPK and Akt results in impaired migration in the EF, which suggests that EGFR, p38 MAPK, and Akt are involved in EF guided HaCaT cell migration. Moreover, inhibition of EGFR resulted in obvious downregulation of the activation of Akt and p38 MAPK. When the activation of p38 MAPK was inhibited, the activation of EGFR was not influenced, while the phosphorylation level of Akt decreased markedly. Blocking Akt activity had no influence on the activation of EGFR or p38 MAPK. The above evidence suggested that EFs may guide HaCaT cell migration through the EGFR/p38 MAPK/Akt pathway.

The endogenous electric field has been proven to play an important role in development, regeneration, wound healing, and tumorigenesis [32]. Experimental documentation has demonstrated that EFs, as guiding signals, often override chemical or topographic cues in cell migration [18]. Clinical studies have shown that applied electrical fields may accelerate wound healing via diverse effects [33]. However, the molecular mechanisms involved in electric field-guided cell migration, especially the signal transduction pathway, are still unclear. Our results demonstrated for the first time that p38 MAPK plays a role in electric field-guided HaCaT cell migration. The cells may transduce electrical signals through the EGFR/p38 MAPK/Akt pathway, which results in directed cell migration through electrical fields.

## 5. Conclusions

In conclusion, our experimental results demonstrated that p38 MAPK is a crucial protein involved in sensing electrical signals and results in directed cell migration in EFs. EFs may guide cell migration through the EGFR/p38 MAPK/Akt pathway. Our studies expended the role of p38 MAPK in cells to response to the endogenous EF, which is one of the important extracellular sensors in development, wound healing, and regeneration. The interpretation of the mechanism underlying how the electric field guides cell migration may facilitate the clinical application of EFs in promoting wound healing.

## Data Availability

The raw data supporting the conclusions of this article will be made available by the authors on request.

## References

[1] Zhao M. (2009). Electrical fields in wound healing-An overriding signal that directs cell migration. Semin. Cell Dev. Biol..

[2] Kim H., Park S., Housler G., Marcel V., Cross S., Izadjoo M. (2016). An Overview of the Efficacy of a Next Generation Electroceutical Wound Care Device. Mil. Med..

[3] Kloth L.C. (2014). Electrical Stimulation Technologies for Wound Healing. Adv. Wound Care.

[4] Cuadrado A., Nebreda A.R. (2010). Mechanisms and functions of p38 MAPK signalling. Biochem. J..

[5] Yu B., Wong M.M., Potter C.M., Simpson R.M., Karamariti E., Zhang Z., Zeng L., Warren D., Hu Y., Wang W. (2016). Vascular Stem/Progenitor Cell Migration Induced by Smooth Muscle Cell-Derived Chemokine (C-C Motif) Ligand 2 and Chemokine (C-X-C motif) Ligand 1 Contributes to Neointima Formation. Stem Cells.

[6] Wu Z., He D., Zhao S., Wang H. (2019). IL-17A/IL-17RA promotes invasion and activates MMP-2 and MMP-9 expression via p38 MAPK signaling pathway in non-small cell lung cancer. Mol. Cell. Biochem..

[7] Xu J., Shi J., Tang W., Jiang P., Guo M., Zhang B., Ma G. (2020). ROR2 promotes the epithelial-mesenchymal transition by regulating MAPK/p38 signaling pathway in breast cancer. J. Cell. Biochem..

[8] Keyse S.M. (1995). Tyrosine kinase inhibition: An approach to drug development. Hum. Exp. Toxicol..

[9] Tolcher A.W., Yap T.A., Fearen I., Taylor A., Carpenter C., Brunetto A.T., Beeram M., Papadopoulos K., Yan L., De Bono J. (2009). A phase I study of MK-2206, an oral potent allosteric Akt inhibitor (Akti), in patients (pts) with advanced solid tumor (ST). J. Clin. Oncol..

[10] Lin F., Baldessari F., Gyenge C.C., Sato T., Chambers R.D., Santiago J.G., Butcher E.C. (2008). Lymphocyte electrotaxis in vitro and in vivo. J. Immunol..

[11] Guan L., Fan P., Liu X., Liu R., Liu Y., Bai H. (2021). Migration of Human Renal Tubular Epithelial Cells in Response to Physiological Electric Signals. Front. Cell Dev. Biol..

[12] Dai L., Zhou J., Li T., Qian Y., Jin L., Zhu C., Li S. (2019). STRIP2 silencing inhibits vascular smooth muscle cell proliferation and migration via P38-AKT-MMP-2 signaling pathway. J. Cell. Physiol..

[13] Wu M., Zhang P. (2020). EGFR-mediated autophagy in tumourigenesis and therapeutic resistance. Cancer Lett..

[14] Huang Y., Wang Y., Wang Y., Wang N., Duan Q., Wang S., Liu M., Bilal M.A., Zheng Y. (2022). LPCAT1 Promotes Cutaneous Squamous Cell Carcinoma via EGFR-Mediated Protein Kinase B/p38 MAPK Signaling Pathways. J. Investig. Dermatol..

[15] Wu D., Ma X., Lin F. (2013). DC electric fields direct breast cancer cell migration, induce EGFR polarization, and increase the intracellular level of calcium ions. Cell Biochem. Biophys..

[16] Zimolag E., Borowczyk-Michalowska J., Kedracka-Krok S., Skupien-Rabian B., Karnas E., Lasota S., Sroka J., Drukala J., Madeja Z. (2017). Electric field as a potential directional cue in homing of bone marrow-derived mesenchymal stem cells to cutaneous wounds. Biochim. Biophys. Acta Mol. Cell Res..

[17] Zhao M., Pu J., Forrester J.V., McCaig C.D. (2002). Membrane lipids, EGF receptors, and intracellular signals colocalize and are polarized in epithelial cells moving directionally in a physiological electric field. FASEB J. Off. Publ. Fed. Am. Soc. Exp. Biol..

[18] Zhao M., Song B., Pu J., Wada T., Reid B., Tai G., Wang F., Guo A., Walczysko P., Gu Y. (2006). Electrical signals control wound healing through phosphatidylinositol-3-OH kinase-gamma and PTEN. Nature.

[19] Fenteany G., Janmey P.A., Stossel T.P. (2000). Signaling pathways and cell mechanics involved in wound closure by epithelial cell sheets. Curr. Biol..

[20] Nuccitelli R. (2003). A role for endogenous electric fields in wound healing. Curr. Top. Dev. Biol..

[21] Tsai H.F., Gajda J., Sloan T.F., Rares A., Shen A.Q. (2019). Usiigaci: Instance-aware cell tracking in stain-free phase contrast microscopy enabled by machine learning. Softwarex.

[22] Beggs J.E., Tian S., Jones G.G., Xie J., Iadevaia V., Jenei V., Thomas G., Proud C.G. (2015). The MAP kinase-interacting kinases regulate cell migration, vimentin expression and eIF4E/CYFIP1 binding. Biochem. J..

[23] Huang C., Jacobson K., Schaller M.D. (2004). MAP kinases and cell migration. J. Cell Sci..

[24] Li F., Chen T., Hu S., Lin J., Hu R., Feng H. (2013). Superoxide mediates direct current electric field-induced directional migration of glioma cells through the activation of AKT and ERK. PLoS ONE.

[25] Lu C., Kolbenschlag J., Nüssler A.K., Ehnert S., McCaig C.D., Čebron U., Daigeler A., Prahm C. (2021). Direct Current Electrical Fields Improve Experimental Wound Healing by Activation of Cytokine Secretion and Erk1/2 Pathway Stimulation. Life.

[26] Cao L., McCaig C.D., Scott R.H., Zhao S., Milne G., Clevers H., Zhao M., Pu J. (2014). Polarizing intestinal epithelial cells electrically through Ror2. J. Cell Sci..

[27] Liu Q., Song B. (2014). Electric field regulated signaling pathways. Int. J. Biochem. Cell Biol..

[28] Jeong D., Lee J., Yi Y.S., Yang Y., Kim K.W., Cho J.Y. (2013). p38/AP-1 Pathway in Lipopolysaccharide-Induced Inflammatory Responses Is Negatively Modulated by Electrical Stimulation. Mediat. Inflamm..

[29] Rouabhia M., Park H.J., Abedin-Do A., Douville Y., Méthot M., Zhang Z. (2020). Electrical stimulation promotes the proliferation of human keratinocytes, increases the production of keratin 5 and 14, and increases the phosphorylation of ERK1/2 and p38 MAP kinases. J. Tissue Eng. Regen. Med..

[30] Tian X., Zhou D., Chen L., Tian Y., Zhong B., Cao Y., Dong Q., Zhou M., Yan J., Wang Y. (2018). Polo-like kinase 4 mediates epithelial-mesenchymal transition in neuroblastoma via PI3K/Akt signaling pathway. Cell Death Dis..

[31] Fang K.S., Ionides E., Oster G., Nuccitelli R., Isseroff R.R. (1999). Epidermal growth factor receptor relocalization and kinase activity are necessary for directional migration of keratinocytes in DC electric fields. J. Cell Sci..

[32] Nuccitelli R. (2003). Endogenous electric fields in embryos during development, regeneration and wound healing. Radiat. Prot. Dosim..

[33] Yang Y., Luo R., Chao S., Xue J., Jiang D., Feng Y.H., Guo X.D., Luo D., Zhang J., Li Z. (2022). Improved pharmacodynamics of epidermal growth factor via microneedles-based self-powered transcutaneous electrical stimulation. Nat. Commun..

